# Experimental evidence and isotopomer analysis of mixotrophic glucose metabolism in the marine diatom *Phaeodactylum tricornutum*

**DOI:** 10.1186/1475-2859-12-109

**Published:** 2013-11-14

**Authors:** Yuting Zheng, Andrew H Quinn, Ganesh Sriram

**Affiliations:** 1 Department of Chemical and Biomolecular Engineering, University of Maryland, College Park, Maryland, MD 20742, USA

**Keywords:** *Phaeodactylum tricornutum*, Glucose, Mixotrophy, Isotope labeling, Metabolic pathway analysis, Entner-Doudoroff

## Abstract

**Background:**

Heterotrophic fermentation using simple sugars such as glucose is an established and cost-effective method for synthesizing bioproducts from bacteria, yeast and algae. Organisms incapable of metabolizing glucose have limited applications as cell factories, often despite many other advantageous characteristics. Therefore, there is a clear need to investigate glucose metabolism in potential cell factories. One such organism, with a unique metabolic network and a propensity to synthesize highly reduced compounds as a large fraction of its biomass, is the marine diatom *Phaeodactylum tricornutum* (Pt). Although Pt has been engineered to metabolize glucose, conflicting lines of evidence leave it unresolved whether Pt can natively consume glucose.

**Results:**

Isotope labeling experiments in which Pt was mixotrophically grown under light on 100% U-^13^C glucose and naturally abundant (~99% ^12^C) dissolved inorganic carbon resulted in proteinogenic amino acids with an average ^13^C-enrichment of 88%, thus providing convincing evidence of glucose uptake and metabolism. The dissolved inorganic carbon was largely incorporated through anaplerotic rather than photosynthetic fixation. Furthermore, an isotope labeling experiment utilizing 1-^13^C glucose and subsequent metabolic pathway analysis indicated that (i) the alternative Entner-Doudoroff and Phosphoketolase glycolytic pathways are active during glucose metabolism, and (ii) during mixotrophic growth, serine and glycine are largely synthesized from glyoxylate through photorespiratory reactions rather than from 3-phosphoglycerate. We validated the latter result for mixotrophic growth on glycerol by performing a 2-^13^C glycerol isotope labeling experiment. Additionally, gene expression assays showed that known, native glucose transporters in Pt are largely insensitive to glucose or light, whereas the gene encoding cytosolic fructose bisphosphate aldolase 3, an important glycolytic enzyme, is overexpressed in light but insensitive to glucose.

**Conclusion:**

We have shown that Pt can use glucose as a primary carbon source when grown in light, but cannot use glucose to sustain growth in the dark. We further analyzed the metabolic mechanisms underlying the mixotrophic metabolism of glucose and found isotopic evidence for unusual pathways active in Pt. These insights expand the envelope of Pt cultivation methods using organic substrates. We anticipate that they will guide further engineering of Pt towards sustainable production of fuels, pharmaceuticals, and platform chemicals.

## Background

The search for robust platform organisms suitable for manufacturing economically valuable compounds such as fuels, commodity chemicals, pharmaceuticals and dietary supplements has increasingly turned to unicellular algae. These eukaryotes naturally synthesize many high-value compounds commonly found in plants whilst also displaying the high growth rates and scale-up characteristics of bacteria and yeast. One model species is the marine diatom *Phaeodactylum tricornutum* (Pt). Apart from their unique capability to incorporate silica into their cell walls [1], diatoms also synthesize copious amounts of lipids. Pt typically produces lipids up to 30% of its dry weight [2], nearly 40% of which is the nutritional supplement ω-3-eicosapentaenoic acid [3,4]. This high lipid content is indicative of significant reductive potential, which has been harnessed through genetic engineering to produce the bioplastic poly-3-hydroxybutyrate (PHB) in large quantities, up to 11% of dry weight [5].

Evidencing the potential of Pt as a cell factory, recent research has suggested or uncovered unique metabolic pathways and combinations of pathways previously unseen in unicellular photosynthetic organisms [6-9]. For instance, sequencing of the Pt genome revealed that 7.5% of the genome is of bacterial origin, suggesting the acquisition of many pathways through lateral gene transfer [6]. An investigation of nitrogen metabolism in Pt determined that this organism operates a urea cycle for nitrogen assimilation, contrasting with the nitrogen-eliminating function of the urea cycle in metazoans. However, despite recent advances in understanding and utilizing Pt, several fundamental biological questions still remain unanswered.

One question that first arose in the late 1950s is whether Pt can metabolize glucose. The answer is relevant for utilizing Pt as a cell factory, because heterotrophic fermentation using simple sugars such as glucose remains the most cost effective bioproduction strategy, largely due to the significant challenges in optimizing photobioreactors and race ponds for phototrophic growth [10-12].

Multiple research groups have reported that Pt cannot grow heterotrophically on glucose in the dark, and that mixotrophic growth on glucose does not noticeably increase growth rates under light [13-15]. On addition of ^14^C glucose to Pt cultures under light and dark conditions minimal radioactivity was observed in the cell extract after 1–48 h, suggesting that Pt may be impermeable to glucose. These experiments give the impression that Pt does not uptake or metabolize glucose. On this basis, Zaslavskaia et al. [16] engineered Pt to express the human glucose transporter protein GLUT1. The transformants exhibited glucose uptake as well as growth on glucose in both light and dark, in contrast to wild type or empty vector control lines that neither grew in the dark, nor appeared to consume glucose.

Conversely, multiple researchers have reported results suggesting that Pt may natively consume and metabolize glucose. For example, one study [17] found that the provision of glucose enhanced the growth rate by 27%, increased the respiration rate by 46% and decreased the net maximum photosynthetic rate by only 3%. This suggests that some glucose was respired for ATP production [17]. A separate study [18] found that the supply of 5 g L^-1^ glucose increased maximum biomass productivity and maximum biomass concentration by 43% and 49%, respectively.

Given this conflicting evidence, the question of glucose metabolism by Pt needs to be addressed by convincing molecular evidence. A unique methodology available for resolving this problem is the isotope labeling experiment (ILE), wherein organic carbon sources such as glucose containing different isotopes (“labels”) of carbon (e.g. ^13^C and ^12^C) are supplied to a cell culture. The incorporation of the labeled carbon source into metabolites and biomass components such as proteinogenic amino acids will produce unique ^13^C-^12^C patterns or isotopomers, which can be detected by measuring mass isotopomer distributions (MIDs) of the metabolites or biomass components. Furthermore, analysis of the isotopomer data by metabolic pathway analysis (MPA) will also enable identification of carbon partitioning and flux through metabolic pathways [19-21].

Apart from determining whether Pt consumes glucose, it is also necessary to identify the metabolic pathways Pt uses to convert glucose to biomass and products. Annotation of the Pt genome revealed two alternate glycolytic pathways of bacterial origin in addition to the conventional reactions of the Embden-Meyerhof-Parnas (EMP) pathway [9]. Of these, the Entner-Doudoroff (ED) pathway splits one molecule of 6-phospho-D-gluconate to one molecule of pyruvate and one molecule of glyceraldehyde-3-phosphate, whereas the phosphoketolase (PPK) pathway coverts one molecule xylulose-5-phosphate to glyceraldehyde-3-phosphate and acetylphosphate, which subsequently forms acetyl-CoA. It is important to resolve the carbon partitioning between these three pathways to develop genetic engineering strategies for improved product yield from organic carbon sources. As an example of the potential benefits of the ED and PPK pathways for cell factories, these pathways have each been utilized to enhance production of PHB in bacteria [22] and yeast [23] by increasing the availability of acetyl-CoA and NADPH for PHB biosynthesis. Therefore, determining the role of the glycolytic pathways in Pt could lead to strategies for enhanced production of PHB and other economically attractive compounds.

This article reports various ILEs and MPA that evidence glucose consumption and metabolism by wild type Pt cultures grown under light. Isotopomer analysis revealed that in mixotrophic cultures receiving glucose and dissolved inorganic carbon as carbon sources, glucose accounted for at least 90% of the carbon assimilated into cellular amino acids, the remaining 10% being derived from dissolved inorganic carbon. Furthermore, MPA revealed that the ED pathway is active in glucose metabolism, and that glycine and serine are largely synthesized from glyoxylate through photorespiratory reactions rather than from the EMP pathway metabolite 3-phosphoglycerate. Additionally, gene expression measurements suggested that glucose transporters may not be regulated to enable glucose uptake, but fructose-bisphosphate aldolase 3 (*Fba3*), a rate-limiting step of the EMP pathway, is transcriptionally activated by light, perhaps to facilitate glucose metabolism.

## Results

**Figure 1:**
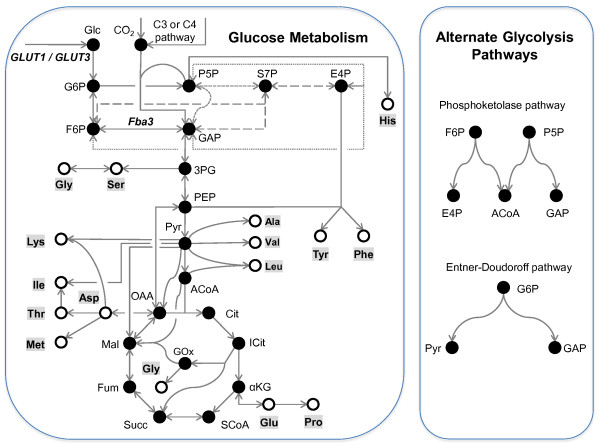
**Principal pathways for the mixotrophic metabolism of glucose and CO**_**2 **_**to amino acids in Pt.** Central carbon metabolic pathways convert glucose and/or CO_2_ (fixed photosynthetically or anaplerotically) to the 15 amino acids (metabolites shown as open circles) experimentally detected by GC-MS in hydrolysates of Pt cell pellets. In most organisms, glycolysis proceeds via the EMP pathway. However, two alternate glycolytic pathways of bacterial origin were found in this organism’s annotated genome. Of these, the phosphoketolase (PKP) enzyme converts phosphorylated pentose and/or hexose sugars to glyceraldehyde 3-phosphate/erythrose 4-phosphate and acetylphosphate, which is then converted to either acetate via acetate kinase, or acetyl-CoA via phosphate acetyltransferase. Both phosphorylated pentose and hexose sugars are shown as substrates for the PPK pathway because the enzyme specificity in Pt is unknown. The second alternative pathway (ED) uses two enzymes to convert 6-phospho-D-gluconate to pyruvate and glyceraldehyde 3-phosphate. Differences in the carbon atom rearrangements of the EMP, PPK and ED pathways become evident in the MIDs of glycolytic amino acids.

To test our hypothesis that Pt metabolizes glucose, we grew Pt cultures for 21 d on media supplemented with 1.917 ± 0.012 g L^-1^ of U-^13^C glucose under both light and dark, and examined if the supplied ^13^C label appeared in biomass components of Pt. In accordance with previously reported results [13-15], cultures kept in the dark exhibited no growth (Additional file 1: Figure S1); therefore, only cultures grown under light were analyzed for ^13^C-enrichment and glucose consumption. Toward this, we harvested biomass at the end of the 21-d steady state ILE (Additional file 2: Figure S2), acid-hydrolyzed the biomass to degrade cellular protein to amino acids and measured the ^13^C enrichments of the amino acids by mass spectrometry (MS). We also analyzed the media and found that the final glucose concentration was 0.791 ± 0.008 g L^-1^, indicating that 59% of the glucose was consumed. Furthermore, on finding evidence of mixotrophic glucose metabolism, we employed MPA to identify the metabolic pathways through which glucose is metabolized. Figure 1 depicts a diagram of potential metabolic pathways including the EMP, the pentose phosphate pathway (PPP), the tricarboxylic acid (TCA) cycle, anaplerosis, glyoxylate shunt and RuBisCO-mediated photosynthesis. The 15 proteinogenic amino acids measured by us are synthesized from precursor metabolites belonging to these pathways; therefore, the labeling patterns in these precursors can be retrobiosynthetically evaluated from those of the amino acids [24]. In our analyses, we also considered the alternative glycolysis pathways (PPK using both hexose and pentose substrates; ED) (Figure 1, right).

## Carbon from U-^13^C glucose appears in proteinogenic amino acids of Pt

**Figure 2:**
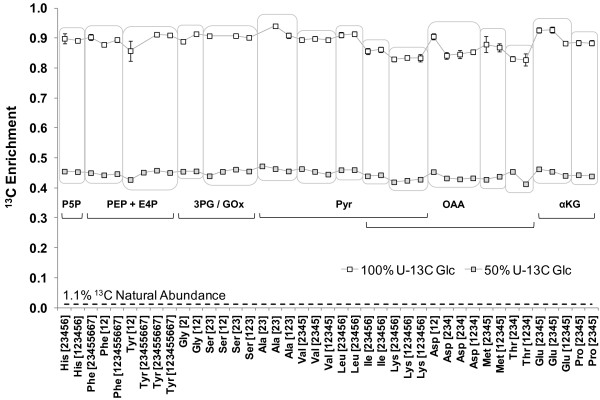
^**13**^**C-enrichments of amino acid fragments synthesized from 100% and 50% U-**^**13**^**C glucose evidence significant glucose uptake.** The 41 measured proteinogenic amino acid fragments in cell hydrolysates of Pt are grouped according to their metabolic precursor(s) (Figure 1). In each of the 100% and 50% U-^13^C glucose ILEs, the fragments show a ^13^C-enrichment approximately proportional to the ^13^C enrichment of the supplied glucose. In contrast, purely photoautotrophic cells would only be ^13^C-enriched to the 1.1% natural abundance CO_2_ from the flask headspace. A 2% dilution by initially present biomass and a combination of anaplerotic and photosynthetic inorganic carbon fixation explain the slightly lower average enrichments than would be expected for cells consuming glucose as their sole carbon source: 88% ± 3% in the 100% U-^13^C glucose ILE and 45% ± 1% in the 50% U-^13^C glucose ILE.

The ILEs on U-^13^C glucose supplied oppositely labeled glucose (100% U-^13^C or 50% U-^13^C) and dissolved inorganic carbon (naturally abundant; hence, 1.1% ^13^C). Therefore, the ^13^C enrichments of amino acid fragments from these experiments can be used as indicators of the extent to which the carbon atoms of glucose were assimilated into the amino acids. Amino acid fragments from Pt cultures grown on 100% U-^13^C glucose were ^13^C-enriched to 88% ± 3% (average across 38 fragments), whereas fragments from cultures grown on 50% U-^13^C glucose enriched to 45% ± 1% (average across 41 fragments) (Figure 2). These enrichments are substantially higher than the 1.1% enrichment expected if Pt solely consumed dissolved inorganic carbon. In fact, these enrichments are close to those expected (100% and 50%) if Pt solely consumed glucose.

In these U-^13^C glucose ILEs, substantial metabolism of glucose can be expected to give a nearly uniform distribution of the ^13^C label throughout the central metabolic network. In support of this, the ratios of the ^13^C enrichments of different amino acid fragments in the 100% U-^13^C:50% U-^13^C glucose ILEs are generally equal to 100%:50% or 2:1. For example, the enrichment ratios of entire amino acid molecules originating in upper glycolysis and the PPP were 89%:45% (histidine) and 89%:45% (phenylalanine). For amino acids originating in lower glycolysis, the ratios were 91%:46% (alanine) and 89%:45% (valine). Amino acids originating in the TCA cycle displayed the ratios 87%:44% (methionine) and 88%:44% (glutamate).

Although a majority of the amino acid fragments showed nearly uniform ^13^C enrichments in the two U-^13^C ILEs, some fragments derived from oxaloacetate and α-ketoglutarate that contained carbon fixed through anaplerotic reactions were enriched to lower extents. Given that the initially present, unlabeled cell mass constituted 2% of the final mass (measurements not shown), the remaining ~10% of unlabeled carbon in the 100% U-^13^C glucose ILE could only have been assimilated from dissolved inorganic carbon, which was ultimately derived from atmospheric, naturally abundant CO_2_. Pt assimilates inorganic carbon through two mechanisms: direct, RuBisCO-mediated photosynthesis or anaplerotic fixation mediated by multiple reactions including phosphoenolpyruvate carboxylase (Zheng Y and Sriram G, unpublished data). The latter mechanism incorporates naturally abundant carbon into oxaloacetate C-4, which is then transferred to α-ketoglutarate C-1 through the TCA cycle. Below, we use an isotopomer notation wherein boldfaced numbers denote ^13^C and numbers in regular font represent ^12^C; thus, glutamate{1**2345**} represents a glutamate isotopomer with ^12^C at C-1 and ^13^C at C-2 to C-5. In our dataset (Figure 2), the anaplerotic mechanism was supported by the higher ^13^C-enrichment of the glutamate{2345} fragment compared to the enrichment of the glutamate{12345} fragment. The abundance of glutamate{1**2345**} as calculated from the MID data by singular value decomposition (SVD) was substantial (22% ± 3%). This indicates that the ^12^C dilution of some amino acids synthesized from TCA cycle metabolites was primarily the result of ^12^CO_2_ incorporation by anaplerotic reactions. In comparison, RuBisCO fixes inorganic carbon through a series of plastidic reactions onto C-1 of valine. The abundance of valine{1**2345**} was significantly lower (3% ± 1%) than that of glutamate, which indicates that during mixotrophic growth on glucose, the anaplerotic reactions assimilate more carbon than the photosynthetic reactions.

### MPA of a 100% 1-^13^C glucose ILE reveals flux through the ED pathway

Our initial MPA focused on constructing a flux map by using a commonly observed set of central carbon metabolic pathways prevalent in most plants and algae [25-27]. This set included the EMP pathway, the PPP, the TCA cycle, RuBisCO-mediated photosynthesis and anaplerotic fixation of unlabeled inorganic carbon. However, the goodness-of-fit for these models, as quantified by the sum of squared residuals (SSR) between the measured MIDs and MIDs simulated by the model, was well above the statistically acceptable threshold. Therefore, we constructed a series of nine metabolic models containing combinations of various catabolic pathways identified in the annotated Pt genome, so that we could identify (a) set(s) of pathways that would account for the experimental isotope labeling patterns. All models were evaluated by using SSR as an acceptability criterion. See Materials and methods for further details.

**Figure 3:**
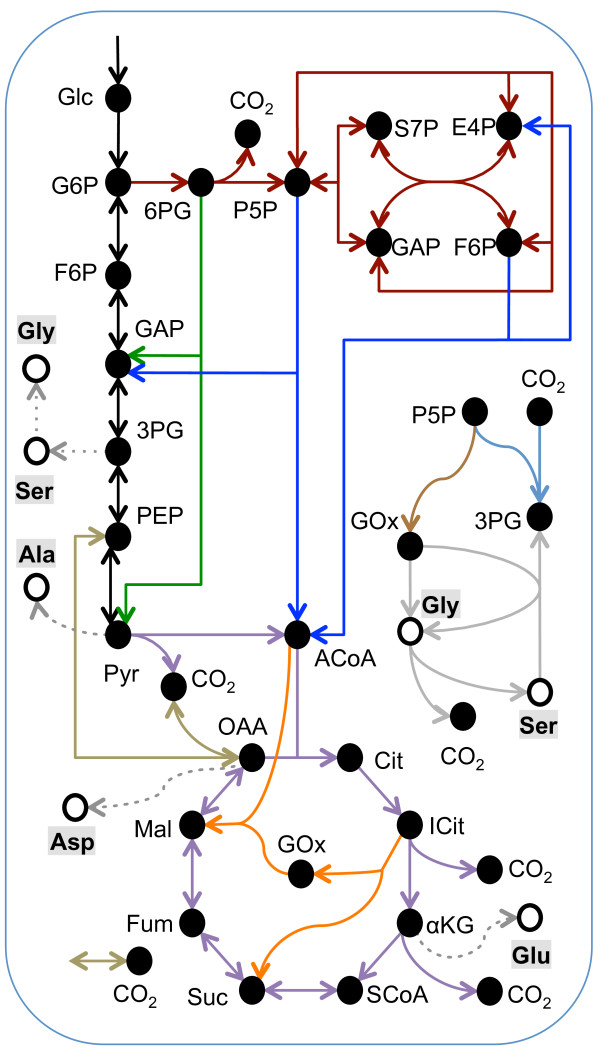
**Pathways used to assemble the metabolic pathway analysis models.** This metabolic network includes 10 metabolic pathways distinguished by line color. Metabolic Models I-IX consist of different combinations of these pathways. Open circles represent amino acids detected in cell hydrolysates, whose isotope labeling patterns were used in the MPA. The amino acids are connected to their metabolic precursor(s) by dotted lines. Genes encoding proteins catalyzing all reactions in these pathways were found in the annotated Pt genome.

**Figure 4:**
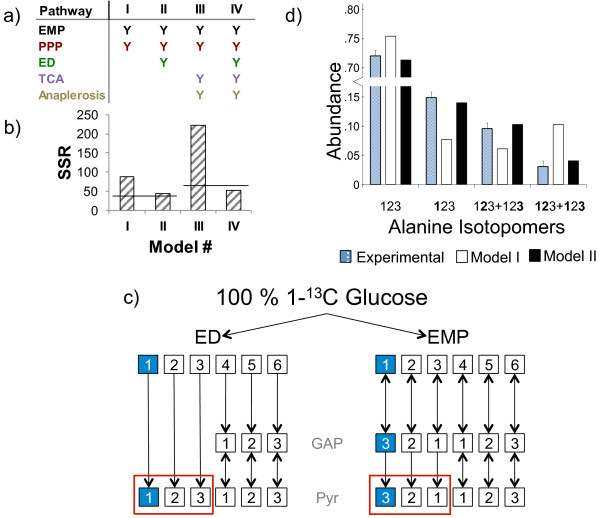
**MPA of the 100% 1-**^**13**^**C glucose ILE data suggests an active ED pathway. (a)** Four different Metabolic Models (I-IV) were constructed to explain the ILE data; each column represents a different model. Pathways included in a model are denoted by a “Y”, color-coded according to the color of the pathway in Figure 3. **(b)** Vertical bars represent the SSR of each model and horizontal lines represent the acceptable SSR corresponding to the number of redundant isotopomer measurements in each model. Models II and IV, both containing the ED pathway show a significantly decreased SSR compared to otherwise identical models lacking the ED pathway. **(c)** The carbon rearrangements of the EMP and ED pathways are shown, with ^13^C atoms shown as blue squares and ^12^C atoms shown as white squares. The EMP pathway transfers ^13^C from glucose C-1 to pyruvate C-3, whereas the ED pathway transfers ^13^C from glucose C-1 to pyruvate C-1 (red boxes). **(d)** The isotopomers of pyruvate reflect those of the amino acid alanine. The measured abundances of alanine isotopomers are compared against the simulated enrichments of Models I and II. Model I that lacks the ED pathway over-simulates the abundance alanine{12**3**}, and under-simulates the abundance of alanine{**1**23}. These errors are corrected in Model II, which utilizes the ED pathway. Isotopomer notation is explained in text.

**Figure 5:**
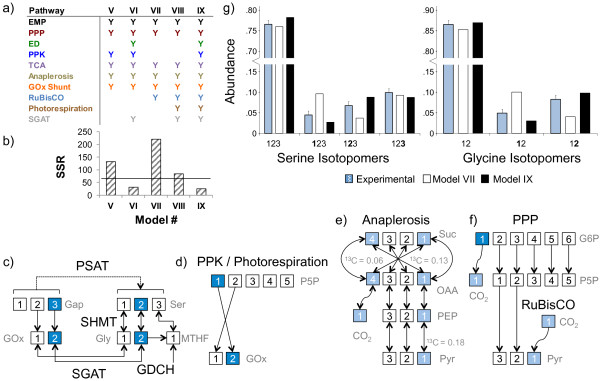
**Unique carbon-carbon bond re-arrangements explain abnormal isotope abundances from the 100% 1-**^**13**^**C Glc ILE. (a)** Five different Metabolic Models (V-IX) were constructed to explain the ILE data; each column represents a different model. Pathways included in a model are denoted by a “Y”, color-coded according to the color of the pathway in Figure 3. **(b)** Vertical bars represent the SSR of each model and horizontal lines represent the acceptable SSR (65) corresponding to the number of redundant isotopomer measurements (45) in each model. **(c)** The carbon rearrangements of SGAT and SHMT demonstrate how 3-^13^C triose phosphates (derived from 1-^13^C glucose through glycolysis) result in 2-^13^C serine and glycine. Serine is conventionally known to be synthesized directly from 3-phosphoglycerate without carbon rearrangements via PSAT shown with the dashed arrow. **(d)** The photorespiratory action of RuBisCO yields glyoxylate from pentose phosphate, whereas the PPK pathway yields acetate that is converted to glyoxylate via the glyoxylate shunt. Each pathway yields 2-^13^C glycine and serine from 1-^13^C pentose phosphate arising from the reductive PPP. **(e)** Anaplerotic fixation of a mixture of intracellular ^12^CO_2_ and ^13^CO_2_ results in 1-^13^C pyruvate through reversible reactions in the TCA cycle. Succinate is a symmetric molecule; therefore C-1 and C-4 are equivalent. Oxaloacetate is 6% ^13^C-enriched at the C-4 position and 13% enriched at C-1. In comparison, pyruvate is 18% enriched at C-1, indicating that anaplerotic fixation cannot fully account for the labeling on pyruvate. **(f)** The oxidative PPP yields ^13^CO_2_ and U-^12^C ribulose 5-phosphate from 1-^13^C glucose-6-phosphate. Photosynthetic fixation of CO_2_ via RuBisCO then results in 1-^13^C pyruvate. **(g)** The predominant isotopomers of serine and glycine that were simulated in the poorly fit Model VII and the well fit Model IX are compared against the experimental abundance of each isotopomer.

We limited our models to simulate the confirmed cytosolic and mitochondrial amino acids from a 100% 1-^13^C glucose ILE, because amongst the glucose labels used, 1-^13^C glucose has a higher information yield than U-^13^C glucose for a network consisting of glycolysis and related pathways [27]. Additionally, we wanted to eliminate errors from amino acids with identical precursors but different MIDs due to differing compartmentalization. The entire metabolic network is shown in Figure 3, with each constituent pathway distinguished by color. Models I-IX contain different combinations of pathways, as specified in Figures 4a and 5a. A simple model, Model I, encompassed only the EMP and PPP pathways. This model simulated the MIDs of serine, glycine and alanine, comprising 22 redundant mass isotopomers. Model I yielded an SSR of 87 (Figure 4b), which is much higher than the statistically acceptable SSR of 37 corresponding to the set of 22 mass isotopomers. The two alanine fragments contributed a majority (58%) of this SSR (Additional file 3: Figure S3), specifically because this model was unable to mimic the high measured abundance of alanine{**1**23}. The inability of the Model I to account for the isotopomer data is evident from the carbon atom rearrangements in Figure 4c (dark blue squares denote ^13^C atoms). Processing of 1-^13^C glucose by the EMP pathway or the PPP results in alanine{12**3**}. In contrast, the ED pathway cleaves the first three carbons of glucose directly to pyruvate yielding the alanine{**1**23} isotopomer that we experimentally observed. Therefore, we extended Model I by incorporating the ED pathway, resulting in Model II. This extension reduced the SSR from 87 in Model I to 42 in Model II (Figure 4b). The improvement of Model II over Model I is evident from a comparison of measured and simulated isotopomers of alanine (Figure 4d).

We further extended Models I and II by including the TCA cycle and anaplerotic fixation of a mixture of atmospheric ^12^CO_2_ and ^13^CO_2_ generated from multiple intracellular decarboxylation reactions. The resulting Models III and IV simulated the MIDs of aspartate and glutamate in addition to alanine, serine and glycine, which summed to 45 redundant mass isotopomers, corresponding to a statistically acceptable SSR of 65. Just as in Models I and II, including the ED pathway in Model IV significantly increased the fit compared to Model III. In this case, the decrease in the SSR from 222 in Model III to 50 in Model IV was due to an increased fit of all the amino acid fragments with the exception of glycine{2} (Figure 4b).

The first four models consistently under-simulated the ^13^C-enrichment of glycine{2}. As shown in Figure 5d, higher enrichments of glycine{2} are possible if a combination of the PPK and the glyoxylate shunt are active. In this situation, ribose-5-phosphate is labeled at C-1 through the transketolase reaction in the PPP/Calvin cycle. The glyoxylate shunt in combination with anaplerotic reactions allows for the C-2 label to reach glycine. Adding the PPK and the glyoxylate shunt in Model V to the previous reactions from Model III partially corrected this error and lowered the SSR in the glycine and serine fragments from 33 to 20 (Additional file 3: Figure S3). Though the SSR decreased from 222 in Model III to 132 in Model V, it was still high in comparison to Model IV. This suggested that while the PPK pathway helps fit the isotopomers of glycine and serine, the ED pathway is important for to achieving an acceptable fit. We assumed in Models I to V that serine and glycine were synthesized from 3-phosphoglycerate; however, they can be synthesized from glyoxylate via alanine-glyoxylate aminotransferase (AGAT) and serine hydroxymethyltransferase (SHMT) as shown in Figure 5c. We dramatically reduced the SSR to an acceptable value of 30 in Model VI by including these reactions along with the ED and PPK pathways.

While Model VI fit the experimental MIDs extremely well, the high ^13^C-enrichment on C-1 of alanine that the ED pathway successfully mimicked could also result from anaplerotic carbon fixation or photosynthesis. This would indicate that Pt generates a significant amount of labeled ^13^CO_2_ intracellularly from decarboxylation reactions, which it then recycles through either of the carbon fixation mechanisms (Figures 5e and 5f; light blue squares represent a mixture of ^12^CO_2_ and ^13^CO_2_). Anaplerotic ^13^CO_2_ fixation manifests on C-1 of glutamate, which was enriched to 6%. This was far less than the 15% enrichment of alanine{**1**23}, indicating that anaplerotic fixation alone could not account for the labeling patterns of alanine. A combination of photosynthetic and anaplerotic fixation was also ruled out by Model VII, which lacked the ED and PPK pathways and yielded a high SSR of 221. We were able to produce a nearly acceptable simulation without the ED or PPK pathways in Model VIII. This model added photorespiration, with serine and glycine synthesized from glyoxylate, to the pathways in Model VII. This lowered the SSR to 84; however, it was still above the acceptable value of 65. Finally, we constructed Model IX with all of the pathways used in previous models. This model yielded nearly identical results as Model VI, with an SSR of only 27.

The average flux values for the three models that met the SSR acceptability criteria (Models IV, VI and IX), calculated from 100 perturbed simulations, diverged due to significant differences in their metabolic pathways. Despite these discrepancies, we noticed a number of trends consistent across all three models. Both the lower and the upper portions of the EMP pathway operated in the reverse direction in nearly all of our simulations, meaning that all of the carbon directed towards acetyl-CoA flowed through the ED or PPK pathways. Table 1 lists the fluxes of the ED pathway, the PPK pathway and the oxidative PPP towards the total flux to acetyl-CoA as well as the ratio of the fluxes through the ED and EMP pathways. The ED pathway contribution to acetyl-CoA synthesis steadily decreased from 100% in Model IV to 33% ± 22% in Model IX, which corresponded to an increase in metabolic cycling as evidenced by the decrease in the ratio of the forward ED pathway flux to the reverse EMP flux from 75% ± 12% in Model IV to 44% ± 13% in Model IX.

**Table 1:** Flux values of reductant-generating pathways from acceptable metabolic models

	**Metabolic Model**		
**Pathway**	**Model IV**	**Model VI**	**Model IX**
ED flux	1.84 ± .90	1.41 ± 0.89	3.85 ± 0.92
PPK flux	N/A	1.71	3.90
Oxidative PPP flux	4.36 ± 0.39	1.60 ± 0.66	1.63 ± 0.78
ED:EMP flux ratio	0.7 ± 0.12	0.69 ± 0.56	0.44 ± 0.13

The metabolic fluxes of the ED, PPK and oxidative PPP pathways were estimated in the three models that met the SSR acceptability criteria, and are reported here in addition to the ratio of the (glycolytic) ED pathway flux versus the (gluconeogenic) EMP pathway flux. Fluxes and ratios are reported as average ± standard deviation, as calculated from distributions generated by 100 bootstrap simulations. All flux values are normalized to an input of 1 mol of glucose. Some flux values are substantially higher than the flux of glucose entering the cell, indicating a metabolic cycle that uses the ED and PPK pathways to produce two and three carbon metabolites, a large fraction of which are then recycled through the reverse EMP pathway. Nevertheless, carbon is strictly conserved in all reactions in our model.

### Glycine and serine are predominantly produced from glyoxylate rather than 3-phosphoglycerate

**Figure 6:**
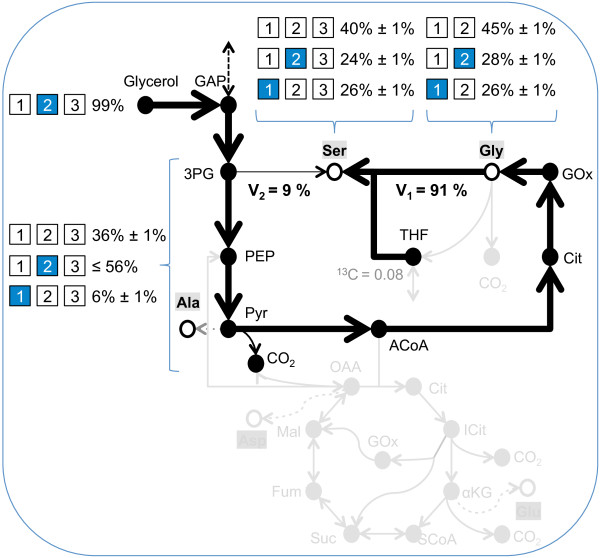
**100% 2-**^**13**^**C glycerol ILE shows that glycine and serine are predominantly synthesized from glyoxylate rather than 3-phosphoglycerate.** Feeding 2-^13^C glycerol to Pt confirmed the MPA prediction that the MIDs of glycine and serine do not represent the labeling of 3-phosphoglycerate as is usual in many organisms. Were this the case, the majority of the ^13^C label from 2-^13^C glycerol would appear on the C-2 of glycine and serine, contradicting observation. The observed isotope labeling patterns in serine and glycine can be explained as follows. First, 2-^13^C glycerol is metabolized to pyruvate and alanine. Carbon rearrangements in the TCA cycle (gray) and back-mixing through anaplerotic reactions and the pentose phosphate pathway account for the small amount of label on alanine{1}. As 3-phosphoglycerate and pyruvate are closely linked to one-another, their MID’s are assumed to be identical. The high abundance of the glycine{**1**2} and serine{**1**23} result from the conversion of pyruvate to acetyl-CoA and glyoxylate. Aminotransferases convert glyoxylate to glycine, which then combines with MTHF to form serine via SHMT. A linear combination of the fluxes from alanine to serine and (glycine + MTHF) to serine produced a set of isotopomers that exactly matched the measured values when the SHMT reaction contributed 91% of the total flux and phosphoserine transaminase contributed 9% of the flux. Arrow widths correspond to relative fluxes. See text for isotopomer notation.

Our models consistently explained isotopomer data if they included a biosynthetic route for glycine and serine from glyoxylate via the photorespiratory reactions AGAT and SHMT. This contrasts with the conventional biosynthesis of glycine and serine from 3-phosphoglycerate. Particularly, the ^13^C enrichments on C-2 of both amino acids was unexpectedly high (Figure 5g). To test if serine and glycine can be synthesized under mixotrophic conditions from glyoxylate in addition to 3-phosphoglycerate, we grew Pt cultures on 100% 2-^13^C glycerol. An examination of carbon atom rearrangements indicated that synthesis of serine from 3-phosphoglycerate via phosphoserine transaminase (PSAT) would yield serine{1**2**3}. Conversely, synthesis from glyoxylate would yield serine{**1**23} due to the loss of pyruvate{1} during decarboxylation to acetyl-CoA and subsequent transamination of glyoxylate to glycine (Figure 6). Isotopomer data from the 2-^13^C glycerol ILE revealed that as would be expected from glycolytic processing of glycerol, the abundance of alanine{**1**23} (6% ± 1%) was much lower than that of alanine{1**2**3} (≤ 56%). However, the abundance of serine{**1**23} (24% ± 1%) was substantially greater than that of alanine{**1**23} and nearly equaled that of serine{1**2**3} (25% ± 1%). We determined the relative contributions of SHMT (V_1_ = 91%) and PSAT (V_2_ = 9%) towards serine biosynthesis by assuming the MID of 3-phosphoglycerate equaled the MID of pyruvate and solving:

$$\begin{array}{ll} \text{serine}{\left\{123\right\}}_{i} & =v_{1}\text{alanine}{\left\{123\right\}}_{i}\\ & \;+v_{2}\text{glycine}{\left\{123\right\}}_{j}\text{MTHF}{\left\{1\right\}}_{k};i\\ & =1...8,j=1...4,k=1,2 \end{array}$$

where the indices *i*, *j*, and *k* denote the individual isotopomers of each metabolite. Although we assumed in our MPA that glycine was the only source of the methyl group transferred to tetrahydrofolate (THF) via glycine decarboxylase (GDCH), there are other methyl group donor reactions that form 5,10-methylene tetrahydrofolate (MTHF) from THF. Therefore, the ^13^C-enrichment of this transferred methyl group was allowed to freely vary along with *V*_1_ and *V*_2_ when we minimized the SSR between the calculated and measured isotopomers of serine. In addition to estimating that 91% of the serine was produced from the photorespiratory reactions, this calculation also estimated that MTHF was only 8% ^13^C-enriched. Since glycine{2} was enriched to 26%, this substantially lower enrichment indicates that compounds other than glycine contribute the majority of the C-1 methyl groups to MTHF.

### Fba3 is upregulated significantly under light while glucose transporters are less sensitive to light and carbon substrates

**Figure 7:**
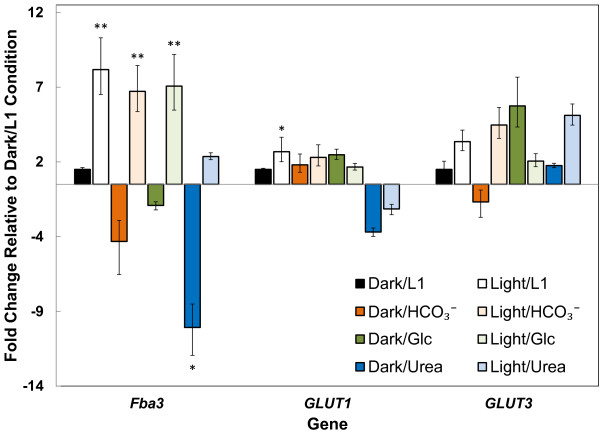
**Expression levels of key glucose-related and glycolytic genes under different environmental conditions.** Expression levels of genes under eight conditions (light/L1, dark/L1, light/HCO_3_^–^, dark/ HCO_3_^–^, light/Glc, dark/Glc, light/urea, and dark urea, see Materials and methods for details), as compared to the dark/L1 condition. Light exposure induces a significant increase in the expression of the cytosolic fructose bisphosphate aldolase 3 gene (*Fba3*) encoding the protein that catalyzes the reversible conversion of fructose 1,6-bisphosphate to glyceraldehyde 3-phosphate and dihydroxyacetone phosphate. The overexpression is independent of the presence of glucose or bicarbonate in the growth media. Conversely, the expression levels of the two genes corresponding to known glucose transporters in Pt (*GLUT1* and *GLUT3*) appear to be unaffected by light exposure and the presence of organic carbon sources. Fold changes were calculated with respect to the housekeeping gene *18S* and were verified with respect to two other housekeeping genes. Results are presented as mean ± SD of three biological and three technical replicates (a total of 9 replicates per gene and condition). *: 0.01 < *p* < 0.05 when compared to the dark/L1 condition; **: *p* ≤ 0.01 when compared to the dark/L1 condition.

By using qRT-PCR, we profiled genes encoding (i) the upper glycolysis enzyme fructose bisphosphate aldolase Fba3 (GenBank 7202915) catalyzing the reversible conversion of fructose-1,6-bisphosphate to glyceraldehyde-3-phosphate and dihydroxyacetone phosphate; and (ii) membrane glucose transporters GLUT1 (GenBank 7198458) *and* GLUT3 (GenBank NC_011676). We performed qRT-PCR on cells in four different media to measure changes in gene expression 1.5 h after switching from light to dark, resulting in eight independent conditions: L1 medium under light (light/L1), L1 medium under dark (dark/L1), HCO_3_^–^-supplemented L1 medium under light (light/HCO_3_^–^), HCO_3_^–^-supplemented L1 medium under dark (dark/ HCO_3_^–^), glucose-supplemented L1 medium under light (light/Glc), glucose-supplemented L1 medium under dark (dark/Glc), urea-supplemented L1 medium under light (light/urea) and urea-supplemented L1 medium under dark (dark/urea). The HCO_3_^–^ and urea samples acted as additional controls to gauge the relative transcription level changes due to light/dark versus changes caused by altering the carbon and nitrogen sources. Figure 7 depicts fold changes with respect to the housekeeping gene *18S* for all genes whose expression levels were consistent across three housekeeping genes (*18S*, *HIS4* and *EF1α*; Additional file 4: Table S14). Clearly, *Fba3* was repressed upon switching from light to dark, irrespective of carbon source supplementation. Exposure to light upregulated *Fba3* by 7.7 ± 1.7-fold (*p* < 0.01) in unsupplemented L1 medium, by 24 ± 2.4-fold (*p* < 0.01) in HCO_3_^–^-supplemented L1 medium, by 9.2 ± 2.1-fold (*p* < 0.01) in glucose-supplemented L1 medium and by 18 ± 1.9-fold (*p* < 0.05) in urea-supplemented L1 medium. Furthermore, urea repressed *Fba3* expression significantly. Under light, *Fba3* expression in urea-supplemented L1 medium was lower by 4.1 ± 0.2-fold (*p* < 0.05) than in unsupplemented L1 medium. Similarly, *Fba3* expression in the dark with urea-supplemented L1 medium was lower by 9.6 ± 1.6-fold (*p* < 0.05) than in unsupplemented L1 medium.

In contrast to *Fba3*, transcription levels of genes encoding glucose transporters did not show consistent trends in light versus dark conditions. The only significant change was the 2.4 ± 1.1-fold (*p* < 0.05) overexpression of *GLUT1* between the light and dark conditions in unsupplemented L1 medium (Figure 7).

In accordance with previous gene expression studies [28], our results show that light transcriptionally activates genes encoding the cytosolic enzyme *Fba3*, which reversibly breaks down fructose 1,6-bisphosphate to three-carbon metabolites in Pt. This suggests that upper glycolysis rather than glucose transportation may be critical to glucose assimilation under light. In addition, urea inhibited expression of *Fba3* in Pt without effecting a significant change on the transcription level of glucose transporters.

## Discussion

One of the goals for developing cell factories is finding flexible organisms that can be rapidly tailored to produce any of a large range of products using the most cost effective substrate available. Unicellular diatoms have the potential to meet this role due to their unique metabolic capabilities and the ease in which they can be genetically manipulated. While Pt has demonstrated a host of advantageous characteristics for cell factories, its utility has been limited by the perception that it cannot consume simple sugars such as glucose.

The ILEs reported in this work have convincingly shown that Pt mixotrophically metabolizes glucose and uses the resulting carbon to synthesize each of the 15 amino acids we measured. As these amino acids are synthesized from multiple nodes encompassing all of primary metabolism across at least three separate intracellular compartments (cytosol, plastid, mitochondrion), it is reasonable to generalize that Pt metabolizes glucose and uses its carbon for the full range of biosynthetic activities. Given this information, it is natural to question why Pt mixotrophically consumes glucose only under light. Our gene expression analysis revealed that transcription level changes of membrane glucose transporters in Pt poorly correlate with exposure to light or glucose. This suggests that the inability of Pt to grow on glucose in the dark is not due to insufficient expression of glucose transporters. However, we cannot discount the possibility that the products of either or both *GLUT1* and *GLUT3* does not transport glucose into the cell, but instead shuttles glucose from the vacuole to the cytosol.

In Pt, light availability has a significant effect over a 24 h period on the expression levels of many genes encoding enzymes in central carbon metabolism [28]. Of the genes encoding glycolysis and glucan biosynthesis, cytosolic *Fba3* is most strongly regulated by light, suggesting that its product may be a rate-limiting enzyme in this pathway. Our gene expression analysis assays confirmed that *Fba3* expression is upregulated by light and further showed that glucose has a negligible regulatory effect under light or dark. Allen et al. [29] showed that of the five fructose bisphosphate aldolase-3 genes in Pt, cytosolic *Fba3* is the only one actively involved in glycolysis and gluconeogenesis, facilitating synthesis of photosynthetically fixed triose phosphates into chrysolaminaran (β:1–3 and β:1–6 glucose polymers) [7]. Our results taken together with previous work on the role of *Fba3*, suggest that in the dark, glucose metabolism is impeded either by the lack of sufficient *Fba3* expression or insufficient transport of glucose into the cell. Our ongoing work is focused on testing the hypothesis that glucose is not transported in the dark by elucidating a light-dependent mechanism for glucose transport and metabolism.

We tested several models of carbohydrate metabolism in Pt. Based upon the SSR criteria, it is clear that an accurate metabolic model includes multiple glycolytic pathways. However, it is certainly possible that other reactions or different combinations of the reactions we chose could produce equally valid results. For example, Model VIII nearly approached the acceptable SSR threshold despite not utilizing the ED and PPK pathways. In this instance, photosynthesis and photorespiration nearly accounted for the unusual observed labeling patterns. If some variation of this model were accurate, it would indicate that Pt uses photosynthesis to re-fix a significant fraction of the CO_2_ that it generates from intracellular reactions, thus conserving organic carbon.

These conflicting possibilities are ordinarily resolved through the use of parallel labeling experiments using large sets of measured metabolites, which greatly increase the confidence intervals of key fluxes and narrow the number of possible pathway models to one or only a small handful of options [30]. However, the high degree of uncertainty regarding the metabolic significance and role of these pathways poses a significant challenge for large-scale ^13^C metabolic flux analysis in Pt. In addition, the compartmentalization of various catabolic and biosynthetic pathways between the cytosol, plastid and mitochondrion is incompletely defined, and the possible effects of metabolic channeling have not been investigated [31]. As a result, we limited our MPA to a single compartment model only including measurements of known cytosolic and mitochondrial amino acids [32]. Due to these simplifications, this article does not report a full quantitative flux map for glucose metabolism using the measurements from multiple ILEs, as was done for multiple bacterial species with active ED pathways [33]. Nevertheless, MPA unraveled much information on the usage of pathways, providing a basis for further developing a quantitative flux map of glucose metabolism in Pt.

MPA suggested that the ED pathway plays an important role in glucose catabolism. Our models consistently showed cycling from both the ED and PPK pathways through the reverse EMP pathway. Such a cycle generates significant excess NADPH that is available for numerous functions including increased lipid production, quenching of reactive oxidative species, and nitrogen assimilation. The traditional oxidative PPP pathway maximally yields 2 moles each of NADPH and NADH per mole of glucose with a 56% carbon yield for lipid synthesis. In comparison, the maximal ED/PPK pathway yields are 1.67/3.33 moles of NADPH and 2.33/0.67 moles of NADH with a matching 67% carbon yield. This hypothesis that Pt can use a combination of the ED and PPK pathways to enhance carbon and cofactor yields for lipid synthesis agrees with the previously reported 17% increase in Pt EPA content when glucose was used in a mixotrophic fed-batch system [34]. Such a metabolic cycle need not be limited to mixotrophic growth on glucose, as the upper reactions in the EMP shuttle carbon in this manner to replenish the supply of 5-C compounds during photosynthesis. Therefore, it is also plausible this metabolic cycling enhances Pt’s ability to synthesize such a high proportion of its biomass as lipids [2-4].

Our gene expression analysis also hints at metabolic cycling, as *Fba3* expression levels were largely unchanged in response to differing carbon sources (dissolved CO_2_, HCO_3_^-^, and glucose), yet changing the nitrogen source from nitrates to urea dramatically inhibited expression in both light and dark conditions. Given that *Fba3* is a key regulator of a major carbon assimilation pathway during photosynthesis, we expected that different carbon sources would have a larger effect on expression levels than changing the nitrogen source. Urea is produced as a consequence of amino acid breakdown, which is then converted to ammonia for nitrogen assimilation. Cells normally synthesize ammonia from nitrates, which requires a substantial supply of reductant. Therefore, urea inhibition of *Fba3* expression may serve to regulate reductant generation.

It is also important to note that the majority of the EMP pathway genes have been found in the cytosol, mitochondria, and plastid; however the gene encoding enolase in the lower half of the pathway has only been identified in the plastid and mitochondria, e.g. from the Diatomcyc metabolic pathway database [9]. Therefore, Pt may lack a complete cytosolic EMP and instead utilize a combination of the ED and PPK pathways to feed the lower half of the mitochondrial EMP.

Another key result we predicted by using MPA and subsequently confirmed through a 2-^13^C glycerol ILE was that serine and glycine are largely derived from glyoxylate instead of 3-phosphoglycerate under some mixotrophic growth conditions. The 2-^13^C glycerol label was used instead of a 2-^13^C glucose label in an attempt to minimize scrambling of the label through the PPP, ED, and upper EMP. Identification of the precursor of serine/glycine could be complicated by PPP carbon atom rearrangements (mostly) and phototrophic ^13^CO_2_ re-fixation. Both these effects can produce 1-^13^C glyceraldehyde 3-phosphate 1-^13^C 3-phosphoglycerate and 1-^13^C serine. However, since pyruvate and alanine are synthesized downstream of 3-phosphoglycerate without net carbon rearrangement, the aforementioned effects should also manifest in the isotopomer distribution of alanine and should generate 1-^13^C alanine with an abundance greater than the abundance of 1-^13^C serine (26%). However, our measurements indicate that the abundance of 1-^13^C alanine is only 6%, substantially less than 26%. Therefore, the caveats of PPP carbon rearrangement and phototrophic ^13^CO_2_ fixation are insufficient to explain the observed 1-^13^C serine, leaving glyoxylate-based synthesis as the only remaining explanation.

These findings are important for future metabolic flux analysis on Pt, as prior work on bacteria and plants grown on glucose has long held that 3-phosphoglycerate [24,35,36] is the sole source of serine and glycine. Further explorations into the mixotrophic synthesis of serine and glycine using multiple organic carbon substrates are warranted, as both AGAT and SHMT are key intermediate reactions in photorespiration and our analysis of the serine and glycine isotopomers indicated that the reactions are active when grown on glycerol. However, the calculated labeling on the transferred methyl group of THF was significantly lower than the labeling on C-2 of glycine, indicating that GDCH is minimally active. Formate is the other major methyl group donor to THF, which is formed as a byproduct of reactions in sterol and cofactor synthesis pathways. Therefore, it is possible that photorespiratory synthesis of glycine and serine is partially due to the need for Pt to recycle THF and clear formate from the cell [37]. Formate is also enzymatically oxidized to CO_2_; however, the reincorporation of formate into serine minimizes carbon loss and should thus be the metabolically favored reaction.

## Conclusions and future work

Our ILEs and analyses convincingly showed that Pt mixotrophically metabolizes both glucose and dissolved inorganic carbon. Specifically, glucose contributes 90% of the carbon assimilated into biomass during exponential growth in batch cultures. MPA provided strong evidence that glucose is metabolized at least partially through the ED pathway, and pinpointed the predominant mechanism for glycine and serine synthesis. Finally, gene expression assays suggested that the cytosolic enzyme Fba3 may be a rate-limiting step in the EMP pathway. Together, our studies resolve a longstanding debate about glucose metabolism in Pt and unraveled the mechanisms through which this sugar is catabolized. We expect that this work will serve as a foundation for future experimental interrogations of diatom metabolism, including an investigation of a unique glucose assimilation mechanism and the possibility of a novel reductant generating pathway via metabolic cycling through parallel glycolytic pathways. This will require an interdisciplinary effort to identify all of the major active pathways in carbon assimilation, unravel their intracellular locations, and understand the metabolic crosstalk between compartmented pathways.

## Materials and methods

### Cell culture and counting

*Phaeodactlyum tricornutum* (strain CCMP 632) was obtained from the Provasoli-Guillard National Center for Marine Algae and Microbiota (NCMA) (East Boothbay, ME), and maintained aseptically by subculturing biweekly. Cultures were grown at 24.5°C under constant light in 125 mL Erlenmeyer flasks containing 50 mL L1 culture medium (NCMA) prepared in sea water (NCMA). Irradiance levels ranged between 40–80 μmol m^-2^ s^-1^ of photons depending on the location of each flask in our shakers as measured using a MQ-100: Quantum integral sensor with handheld meter (Apogee Instruments) (Logan, UT). No changes in algal growth rates were observed across this range of light intensities (data not shown). The flasks were placed in refrigerated New Brunswick Innova 44R shakers (Eppendorf, Hauppauge, NY) with a 2-inch stroke and programmable temperature, light and photoperiod controls. Flasks were sealed with a porous foam stopper to prevent contamination and allow free exchange of air. Cell numbers were measured daily by aseptically transferring small aliquots (10 μL) of cell suspension from cultures to INCYTO C-Chip disposable hemacytometers (ThermoFisher Scientific, Waltham, MA) and counting visible cells with an Axiovert 135 TV microscope (Zeiss Oberkochen, Germany) at 20X resolution. Three biological replicates each with two technical replicates were counted for each time point. During later stages of growth, cell suspensions were diluted in sea water to prevent overcrowding and to maintain cell densities at less than ~100 cells per chip. Glucose measurements were performed in triplicate by pipetting ~1 ml of media into a 2 ml microcentrifuge tube, and inserting the tube into YSI 2300 STAT Plus Glucose & Lactate Analyzer (YSI Life Sciences) (Yellow Springs, OH).

### Gene expression analysis by quantitative real-time polymerase chain reaction (qRT-PCR)

Cells were grown as described in the previous paragraph for 9 d to obtain sufficient biomass. The biomass was divided into eight groups: (i) dark/L1, (ii) light/L1, (iii) dark/HCO_3_^–^ (iv) light/ HCO_3_^–^, (v) dark/glucose, (vi) light/glucose, (vii) dark/urea and (viii) light/urea [same order as in figure], with each condition being represented by three biological replicates. The flasks in the L1 groups (L1) were incubated in (50 mL of) L1 medium, whereas the flasks in the HCO_3_^–^, glucose (Glc) and urea (Urea) groups were incubated in (50 mL of) L1 medium supplemented aseptically with 0.5 mL of 33 g L^-1^ NaHCO_3_ solution, 2 g L^-1^ glucose solution and 3.7 g L^-1^ urea, respectively. After incubation for 14 h under constant light and normal growth conditions, the flasks in the dark/L1, dark/ HCO_3_^–^, dark/glucose and dark/urea groups were transferred to complete darkness and incubated for 90 min. Following this, the cell suspension from each flask was centrifuged at 8000 min^-1^ for 5 min. The wet cell pellets, suspended in less than 0.5 mL medium, were transferred to separate 2 mL sterilized micro-centrifuge tubes, which were quenched immediately with liquid nitrogen. RNA was extracted by using RNeasy Plant Mini Kits and RNase-Free DNase Set (QIAGEN, Valencia, CA). RNA concentrations in the extracts were quantified with a NanoDrop 2000 UV–vis spectrophotometer (Thermo Scientific). cDNA was synthesized from RNA using a High Capacity RNA-to-cDNA Kit (Life Technologies, Grand Island, NY) and random primers. qRT-PCR analyses were conducted with Power SYBR Green PCR Master Mix (Life Technologies) on a 7500 Real-Time PCR System (Life Technologies). The genes encoding 18S rRNA (*18S*), histone 4 (*HIS4*) and elongation factor 1α (*EF1α*) were used as housekeeping genes [38]. The gene-specific primers used for amplification are listed in Additional file 4: Table S14. The three biological replicates for each condition were each analyzed three times. For each of the eight conditions tested, gene expression fold changes relative to the dark/L1 condition were obtained by using the 2^-*ΔΔCt*^ method [39], and statistical significance was determined by using a Student’s *t*-test.

### Mixotrophic ILEs, cell harvest, protein extraction, hydrolysis and derivatization

Steady-state, mixotrophic ILEs were performed by adding one of 100% U-^13^C glucose, 50% U-^13^C glucose, 100% 1-^13^C glucose, or 100% 2-^13^C glycerol to L1 medium. Only one isotopically labeled substrate was added in each experiment. The addition was performed aseptically before subculturing so as to result in the final concentration of 2 g L^-1^ of substrate. Each mixotrophic ILE was represented by 3 to 4 biological replicates. Additionally, the 100% U-^13^C glucose experiment was repeated with matching results (data not shown) on a second cell line of the identical strain of Pt purchased from the NCMA. Cells from the mixotrophic ILEs were harvested at 21 d of culture. Evidence supporting the establishment of isotopic steady state at this time point is shown in Additional file 2: Figure S2. The cell suspensions were centrifuged at 8000 min^-1^ for 30 min, and the supernatant was removed. The cell pellet was briefly resuspended in 50 mL deionized water to rinse out salts and then centrifuged again, after which the supernatant was removed. Cellular metabolism was quenched by immersing tubes containing the pellets in liquid nitrogen. The quenched cells were lyophilized overnight at room temperature and 133 μbar. The lyophilized pellet was hydrolyzed by adding 3 mL 6 N HCl and incubating at 155°C for 4 h to obtain proteinogenic amino acids. Before hydrolysis, the hydrolysis tube was evacuated, then flushed with nitrogen to remove residual oxygen, and then re-evacuated, followed by two more repetitions of these steps. The resulting hydrolysate was cooled to room temperature, filtered by glass wool and dried overnight in a RapidVap evaporator (Labconco, Kansas City, MO) at 55°C, 80 mbar. The dried sample was mixed with deionized water and lyophilized again. After lyophilization, this mixture was reconstituted in 200 μL dimethylformamide (DMF) and derivatized with 80 μL N-(tert-butyldimethylsilyl)-N-methyltrifluoroacetamide MTBSTFA + 1% tert-butyldimethylchlorosilane (TBDMCS) (Thermo Scientific, Rockford, IL) at 70°C for 1.5 h. The derivatized sample was injected into a gas chromatograph (GC)-MS, using DMF as solvent.

### Quantification of mass isotopomer abundances by GC-MS

All GC-MS analyses were performed on a Varian 300MS quadrupole GC-MS unit (Bruker Corporation, Fremont, CA), equipped with an autoinjector and a VF5-ms column of dimensions 0.25 mm × 30 m × 0.25 μm. Typically, 1 μL of derivatized amino acids, in 3 technical replicates, was automatically injected at a split ratio of 1:15, with helium as the carrier gas at a constant flow rate of 1.0 mL min^-1^. The oven temperature was initially held at 150°C for 2 min, then increased at 3°C min^-1^ to 250°C and then at 10°C min^-1^ to 275°C, where it was held constant up to a run time of 43 min. The MS ran in electron ionization mode with a collection delay for 3 min. Mass spectra were recorded in the selected ion monitoring (SIM) mode. All mass spectral data were analyzed and quantified with the manufacturer’s Varian MS Workstation software (Bruker, Billerica, MA). Raw mass spectral data were processed to filter out natural abundances of elements other than metabolic carbon, using a previously developed in-house MATLAB program [see Additional files of 40], whose accuracy has been verified by us by processing a variety of amino acid isotopomer mixtures of known isotopomeric compositions (data not shown). The resulting mass isotopomer distribution data were converted to ^13^C enrichments of individual amino acid fragments by using SVD. The accuracy of the SVD method for obtaining ^13^C enrichments was verified by processing a synthetic set of amino acid MIDs and ensuring that the predicted enrichments were obtained (G. Sriram, unpublished calculations). MIDs obtained from steady-state ILEs are listed in Additional file 4: Tables S1 to S4. Selected MIDs and ^13^C enrichments are shown and discussed in Results. The MIDs were adjusted to account for the presence of initially present unlabeled material that was used to inoculate each flask, so that the MIDs would reflect their true values if no unlabeled material were present. The corrected isotopomers were calculated (data not shown) using the equation *C*_*i*_ = (M_*i*_ –D*NA)/(1-NA_*i*_) for each mass isotopomer *i=0:n* where n is the number of carbon atoms, C is the corrected value, M is the measured value, NA is the natural abundance of that isotopomer, and D is the dilution factor from initially present material. The amount of initially present material was calculated as the ratio of the number of cells on day zero over the number of cells on the final day (data not shown). The calculation of a single dilution factor for all amino acids using the initial and final cell numbers is valid as long as both the weight percent of protein and the amino acid composition do not vary during the experiment. We calculated new dilution factors assuming the mass percent of protein could vary ±50% over the timeframe of the experiment and we found that the new mass isotopomers fell within the standard deviations of our original calculations. We further analyzed the mass spectra of Pt cells grown on multiple substrates and found that changes in the percent composition of the amino acids again yielded smaller changes in the MID’s than the standard deviations of our measurements.

### Evaluation of metabolic fluxes from steady-state isotopomer data

We used our computer program NMR2Flux + [26,27,40] to evaluate and compare the nine different pathway models using corrected MIDs from the steady-state 100% 1-^13^C glucose ILE. The program employs cumomer balancing to simulate ILEs, a simulated annealing-based global optimization algorithm to evaluate fluxes from MS- and NMR-derived isotopomer abundances and a bootstrap Monte Carlo algorithm [41] to evaluate standard deviations or confidence intervals of fluxes. The SSR of each model is calculated with a lower limit on the standard deviation of each mass isotopomer set at 0.01 to account for imprecision in the MS and unknown ^13^C kinetic isotope effects [42]. SSR values are deemed acceptable if they fit a normal χ^2^-distribution, with the degrees of freedom equal to the sum of the redundant mass isotopomers. The metabolic models used by us for flux evaluation from the 100% 1-^13^C glucose ILE are listed in Additional file 4: Tables S5 to S13. The extracellular CO_2_ incorporated through anaplerotic and photosynthetic fixation was labeled to the natural carbon abundance of 1.1% in all of the simulations. Biomass effluxes were taken from the literature for the amino acid [43] lipid and starch composition [44] under phototrophic conditions. Metabolite effluxes, scaled per mole of glucose consumed, were allowed to vary ±50% for the lower and upper bounds in the models in order to account for potential variations in biomass composition. The biomass effluxes are listed in Additional file 4: Table S15.

## Abbreviations

3PG: 3-phosphoglycerate (used in figures); 6PG: 6-phosphogluconate (used in figures); αKG: α-ketoglutarate (used in figures); ACoA: Acetyl-CoA (used in figures); Cit: Citrate (used in figures); E4P: Erythrose 4-phosphate (used in figures); ED: Entner-Doudoroff; EMP: Embden-Meyerhof-Parnas; F6P: Fructose 6-phosphate; Fum: Fumarate (used in figures); G6P: Glucose 6-phosphate (used in figures); GAP: Glyceraldehyde 3-phosphate and dihydroxyacetone phosphate (used in figures); GC-MS: Gas chromatography–mass spectrometry; Glc: Glucose (used in figures); GOx: Glyoxylate (used in figures); Icit: Isocitrate (used in figures); ILE: Isotope labeling experiment; Mal: Malate (used in figures); MID: Mass isotopomer distribution; MPA: Metabolic pathway analysis; MTHF: 5,10-methylene tetrahydrofolate; OAA: Oxaloacetate (used in figures); P5P: Ribose 5-phosphate, pentose 5-phosphate or xylose 5-phosphate (used in figures); PEP: Phosphoenolpyruvate (used in figures); PHB: poly-3-hydroxybutyrate; PPK: Phosphoketolase; PPP: Pentose phosphate; Pt: Phaeodactylum tricornutum; S7P: Sedoheptulose 7-phosphate (used in figures); Pyr: Pyruvate (used in figures); SCoA: Succinyl-CoA (used in figures); SSR: Sum of squared residuals; SVD: Singular value decomposition; Succ: Succinate (used in figures); THF: Tetrahydrofolate; : Amino acids are referred to by their three-letter abbreviations.

## Competing interests

The authors declare that they have no competing interests.

## Authors’ contributions

GS and YZ conceived this study; GS, YZ and AHQ designed it. YZ and AHQ performed the experiments; AHQ and YZ interpreted and analyzed data. AHQ, YZ and GS wrote the manuscript and prepared a revised version. All authors approved the final version.

## Supplementary Material

Additional file 1: Figure S1 **Cell counts evidence that Pt grows on glucose under light but not under dark.** Pt cells were grown on L1 media supplemented with 2 g L^-1^ glucose were sampled and counted on a hemacytometer over a 13 d growth period. Cell numbers increased exponentially for the first 10 d under continuous light (open circles), but did not increase under continuous dark (closed circles). “*” represents statistically significant differences between light-grown and dark-grown cells at the same time point with *p* < 0.05. Click here for file

Additional file 2: Figure S2 **Evidence for isotopic steady state from 20–22 d.** This analysis of Pt cells grown on 100% U-^13^C glucose for 20, 21, and 22 d shows that the MID’s of the amino acid fragments remain nearly constant immediately before and after the standard harvesting time of 21 d. (a) A principal component analysis of 200 mass isotopomers from 38 amino acid fragments using a control sample at time zero and two biological replicates at each time-point shows that the 1^st^ principle component explains 88% of the variance. The abundance of [M + 0], [M + n-1], and [M + n] mass isotopomers of key fragments of aspartic acid and glutamic acid (b) and serine and alanine (c) are plotted from time zero to 22 d. The abundances are noticeably different from time zero to 20 d, but remain constant over the following two days. Click here for file

Additional file 3: Figure S3 **Errors contributed by amino acid fragments to SSR in various MFA Models I to IX.** This heat map depicts the goodness-of-fit SSR criterion for Models I-IX, broken down by amino acid fragment. SSR is representative of the error between the measured mass isotopomers and their simulated values from a particular model; thus, SSR quantifies how well a model accounts for the measured isotope labeling patterns. As shown in the legend, the intensity of red color is proportional to the SSR: darker shades indicate higher SSR and hence a poor fit. Boxes filled with a hashed pattern indicate fragments that were not simulated by that model. On comparing the different fragments (rows), it is clear that some fragments such as glycine{12} and serine{12} are easily fit by all models, whereas others such as glycine{12} and serine{12} are only fit by a few of the models. Click here for file

Additional file 4 **Supplementary data tables S1 to S15. Tables S1 to S4.** MIDs measured in 100% U-^13^C glucose ILE (S1), 50% U-^13^C glucose ILE (S2), 100% 1-^13^C glucose ILE (S3) and 100% 2-^13^C glycerol ILE (S4). We obtained MIDs from GC-MS analysis samples prepared from cell pellet hydrolysates, after correcting for natural abundances of elements other than metabolic carbon (see Materials and methods). A few amino acids could not be detected in certain spectra or produced obviously erroneous MIDs due to their low abundance; the corresponding MIDs are marked as not determined (nd). **Table S5 to S13.** Stoichiometries and carbon atom rearrangements for Metabolic Models I-IX used for flux evaluation from the 100% 1-^13^C glucose ILE data. The columns titled 'DiatomCyc’ and 'KEGG’ refer to the two annotated genome databases for Pt [9]. The color-coded circles indicate whether genes coding for the proteins catalyzing the reaction(s) listed in the row are identified in the database. Some reactions in the models condense multiple metabolic reactions into a single step; therefore green circles indicate that all reactions have corresponding genes, yellow circles indicate that some reactions have corresponding genes, and red circles indicate that none of the reactions have corresponding genes. **Table S14.** Primers used for qRT-PCR. **Table S15.** Biomass efflux values for Metabolic Models I-IX. The lower bounds (LB) and upper bounds (UB) deviate 50% from the calculated value. Click here for file
