# Supplementary figures and images for: Experimental evidence and isotopomer analysis of mixotrophic glucose metabolism in the marine diatom Phaeodactylum tricornutum
Source: Microb Cell Fact. 2013 Nov 14;12:109. doi: 10.1186/1475-2859-12-109 (PMC3842785; doi:10.1186/1475-2859-12-109)

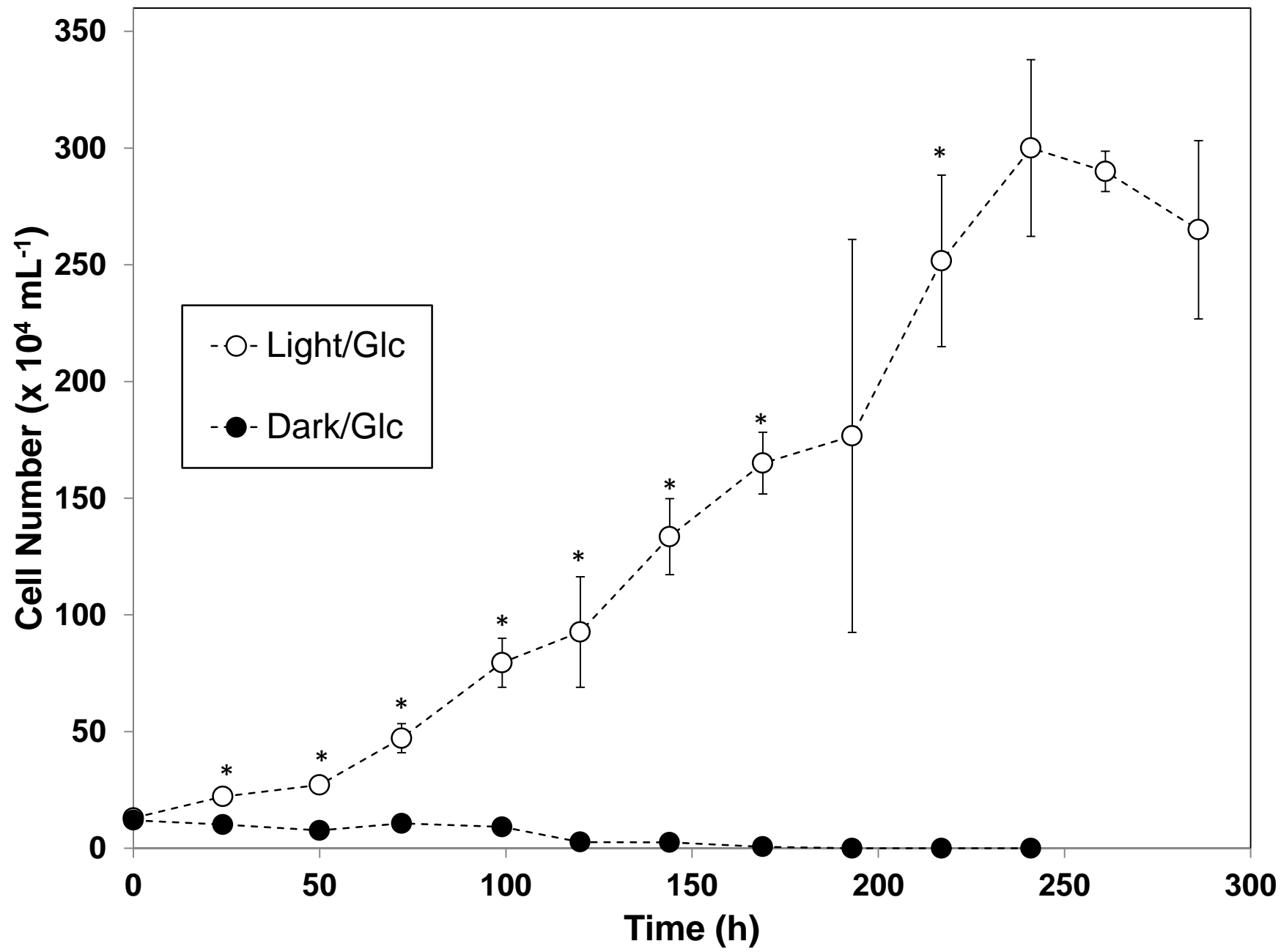

Supplement: Additional file 1: Figure S1 — Cell counts evidence that Pt grows on glucose under light but not under dark. Pt cells were grown on L1 media supplemented with 2 g L-1 glucose were sampled and counted on a hemacytometer over a 13 d growth period. Cell numbers increased exponentially for the first 10 d under continuous light (open circles), but did not increase under continuous dark (closed circles). “*” represents statistically significant differences between light-grown and dark-grown cells at the same time point with p < 0.05. [file 1475-2859-12-109-S1.pdf]

a)

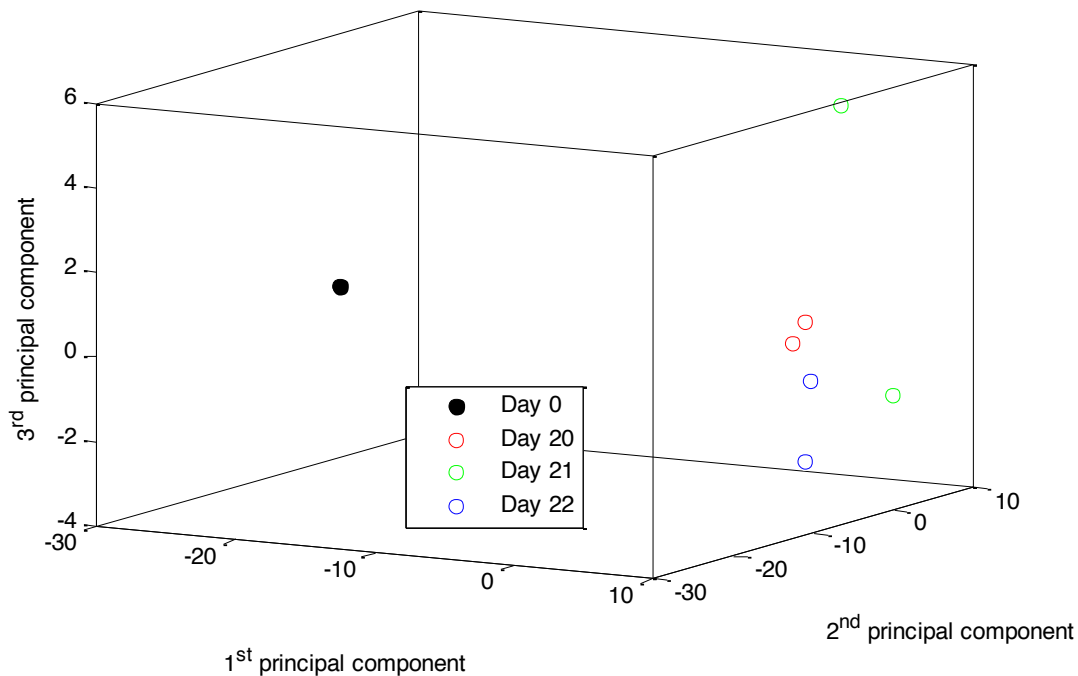

b)

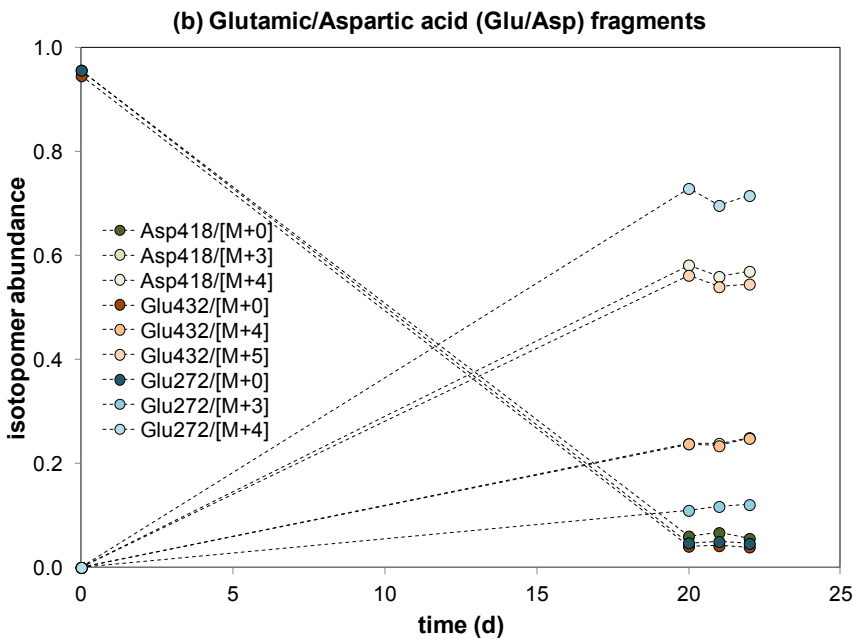

c)

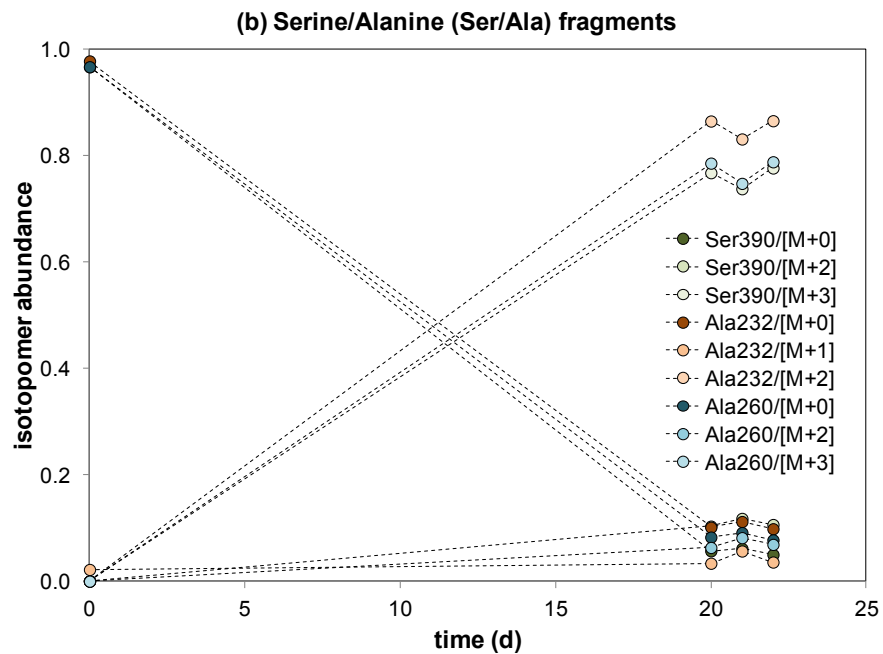

Supplement: Additional file 2: Figure S2 — Evidence for isotopic steady state from 20–22 d. This analysis of Pt cells grown on 100% U-13C glucose for 20, 21, and 22 d shows that the MID’s of the amino acid fragments remain nearly constant immediately before and after the standard harvesting time of 21 d. (a) A principal component analysis of 200 mass isotopomers from 38 amino acid fragments using a control sample at time zero and two biological replicates at each time-point shows that the 1st principle component explains 88% of the variance. The abundance of [M + 0], [M + n-1], and [M + n] mass isotopomers of key fragments of aspartic acid and glutamic acid (b) and serine and alanine (c) are plotted from time zero to 22 d. The abundances are noticeably different from time zero to 20 d, but remain constant over the following two days. [file 1475-2859-12-109-S2.pdf]
