# Supplementary material for: Experimental evidence and isotopomer analysis of mixotrophic glucose metabolism in the marine diatom Phaeodactylum tricornutum
Source: Microb Cell Fact. 2013 Nov 14;12:109. doi: 10.1186/1475-2859-12-109 (PMC3842785; doi:10.1186/1475-2859-12-109)
Supplement: Additional file 3: Figure S3 — Errors contributed by amino acid fragments to SSR in various MFA Models I to IX. This heat map depicts the goodness-of-fit SSR criterion for Models I-IX, broken down by amino acid fragment. SSR is representative of the error between the measured mass isotopomers and their simulated values from a particular model; thus, SSR quantifies how well a model accounts for the measured isotope labeling patterns. As shown in the legend, the intensity of red color is proportional to the SSR: darker shades indicate higher SSR and hence a poor fit. Boxes filled with a hashed pattern indicate fragments that were not simulated by that model. On comparing the different fragments (rows), it is clear that some fragments such as glycine{12} and serine{12} are easily fit by all models, whereas others such as glycine{12} and serine{12} are only fit by a few of the models. [file 1475-2859-12-109-S3.pdf]

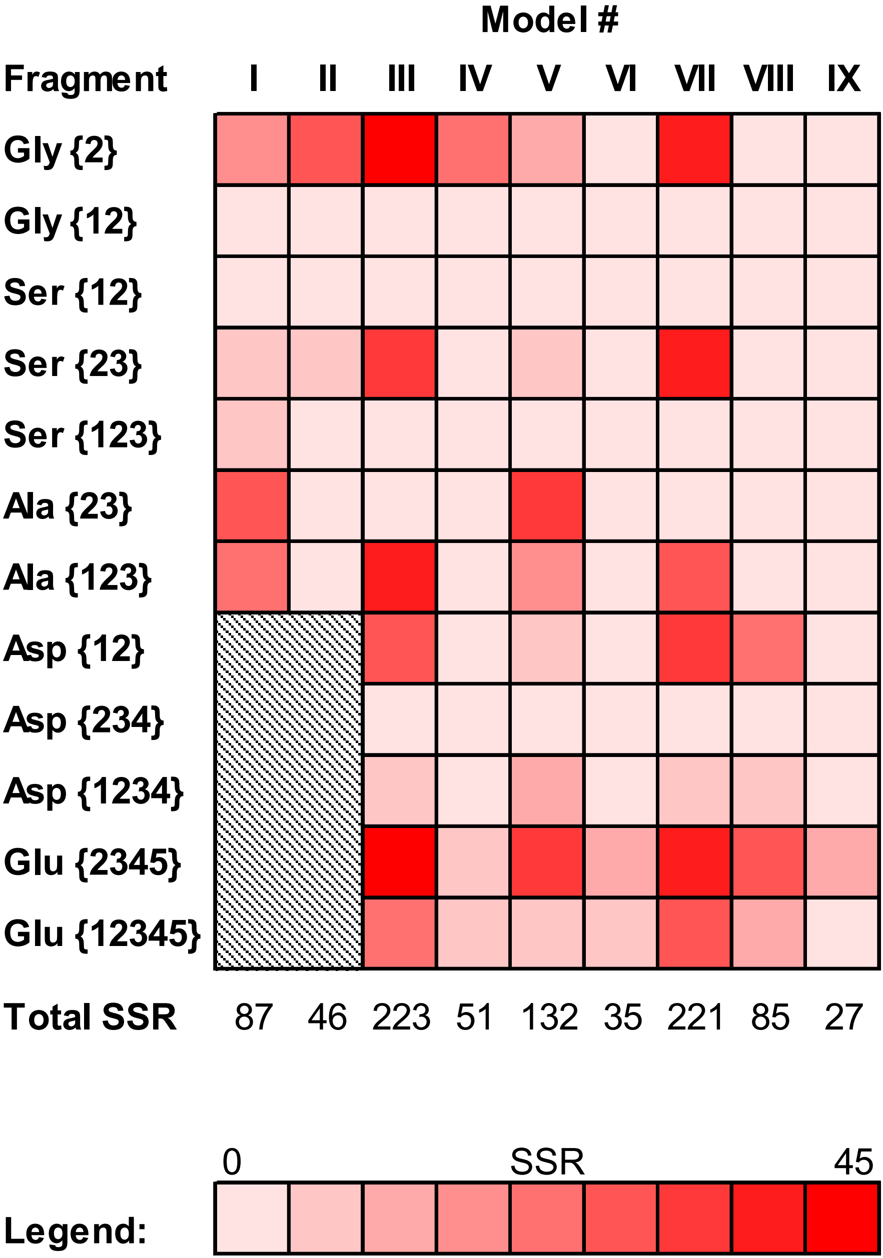
Model #
Fragment
I
II
III
IV
V
VI
VII
VIII
IX
Gly {2}
Gly {12}
Ser {12}
Ser {23}
Ser {123}
Ala {23}
Ala {123}
Asp {12}
Asp {234}
Asp {1234}
Glu {2345}
Glu {12345}
Total SSR
87
46
223
51
132
35
221
85
27
0
SSR
45
Legend:
